# Current Insights into the Neurotoxicity of Melamine: A Comprehensive Review

**DOI:** 10.2174/1570159X22666240320133241

**Published:** 2024-04-08

**Authors:** Reza Naeimi, Fatemeh Safarpour, Hamid Askari, Maryam Ghasemi-Kasman

**Affiliations:** 1 Student Research Committee, Babol University of Medical Sciences, Babol, Iran;; 2 Cellular and Molecular Biology Research Center, Health Research Institute, Babol University of Medical Sciences, Babol, Iran;; 3 Department of Physiology, Faculty of Medicine, Babol University of Medical Sciences, Babol, Iran

**Keywords:** Melamine, neurotoxicity, long term potentiation, ionic currents, oxidative stress, autophagy

## Abstract

Melamine, a heterocyclic nitrogen-rich triazine chemical compound, is widely used in various household products, including furniture, dinnerware, and kitchen appliances. The unauthorized addition of the mixture to various foodstuffs to misrepresent protein content resulted in catastrophic, frequently life-threatening health consequences for kids as well as canines and has garnered international attention. Numerous primary studies and evaluations have been focused on melamine toxicity's implications on kidney function. Despite the profusion of literature on melamine's nephrotoxicity, evidence regarding its toxicity to other organs remains scarce. A number of recent studies suggest melamine can disrupt central nervous system (CNS) function and bring about cognitive impairments, contradicting the commonly held belief that melamine's detrimental effects are limited to the urinary system. The accumulation of melamine in the body is linked to various adverse effects, including depression, impaired synaptic transmission, oxidative stress, and neurodegenerative diseases. Several mechanisms may lead to such complications. However, numerous safeguards against melamine accumulation have been identified. This review could shed light on the potential neurological effects and mechanisms underlying melamine toxicity. Afterward, we will dive into the body's possible protective mechanisms against melamine-induced toxicity.

## 1 INTRODUCTION

Melamine (2,4,6-triamino-s-triazine), a highly stable organic white crystalline powder with a six-membered carbon-nitrogen ring and three amino groups (-NH_2_), has been utilized to synthesize formaldehyde resins to produce thermostable plastics [1, 2]. This chemical is widely used in the manufacturing process of fertilizers, laminates, resins, adhesives, plywood, flooring, paint pigments, and local furniture, such as utensil-making tools [3, 4]. There are numerous routes of entry for melamine into the body. Multiple exposures of melamine containers to microwave heat or the consumption of acidic foods, for instance, may result in melamine and formaldehyde release and intestinal absorption [5].

As melamine, like amino acids, contains substantial quantities of nitrogen, the intentional adulteration of food generates a high rate of false positives in the standard apparent protein content test [6]. Since it was initially added to Chinese infant milk and pet foods, bringing extreme impairments and even mortality, it has received considerable food safety attention [1]. Based on available scientific evidence, the U.S. Food and Drug Administration (FDA) has established a maximum allowable level of melamine in infant formula of 2.5 parts per million (ppm) and a limit of 1 ppm for all other foods [7]. Infants are considered to be more susceptible to the effects of melamine due to their smaller body size, lower renal clearance, more prolonged exposure to melamine and its metabolites, and developing physiological systems. Therefore, they may be more susceptible to the adverse consequences of melamine at lower concentrations than adults [8]. Melamine is typically eliminated from the body within 24 hours following ingestion [9]. However, animal studies indicate that it might accumulate in the uterus, liver, testes, stomach, and spleen of rodents [5]. Even detrimental deposits in some brain regions, such as the hippocampus, cortex, striatum, and cerebellum, may promote cell death [10, 11]. When combined with cyanuric acid, melamine disrupts the membrane of erythrocytes and diminishes the immune systems of youngsters [10].

The substance has also been added to agricultural insecticides containing Cyromazine, which may penetrate the body and degrade into cyanuric acid and melamine [5]. A combination of melamine with uric acid, matrix proteins, and phosphate [12] has been demonstrated to trigger the formation of crystals in the kidney, which might lead to other issues, such as kidney stones. Depending on the dose of melamine ingested, the gender, and the quantity of water consumed [13], renal impairment may ensue. It has been stated that insoluble melamine-cyanurate complexes drive the cell membrane to end up brittle or deformed, thereby affecting energy metabolism [14]. Furthermore, melamine exposure leads to neurotoxic consequences in organisms, involving inhibition of synaptic transmission [15] and oxidative damage [16], as will be discussed in further detail. Likewise, melamine may pass through the placenta in pregnant women, followed by reaching the growing embryo's circulation, inducing histopathological changes, inflammatory reactions, and ultimately fetal death [9]. Prenatal, along with postnatal chronic melamine exposure, could result in cognitive deficits in animal studies [17]. Other symptoms of melamine toxicity include anorexia, vomiting, lethargy, polyuria and polydipsia, high blood urea nitrogen levels, and creatinine levels, which indicate renal failure [18].

## 2 MELAMINE CRYSTALS

Melamine-related nephrolithiasis is uncommon and usually caused by severe or chronic exposure or purposeful food adulteration. Melamine and its derivatives can crystallize in the urinary system in the distal renal nephrons causing urolithiasis, which may cause tubular epithelial necrosis and acute kidney disease (AKD) [19]. Several risk factors may cause urinary tract melamine crystals, including prolonged Melamine overexposure, Insufficient Hydration, acidic urine due to certain drugs and medical conditions, renal Impairment, and low renal clearance. Male gender and low pH are two major risk factors for crystal formation [9, 13]. Intriguingly, increasing the sodium content of the melamine-treated animal's diet increases water consumption, thereby decreasing the formation of kidney stones [13].

Hematuria, crystalluria, urolithiasis, nephrolithiasis, epithelial hyperplasia, and kidney and bladder stones are the most prevalent clinical signs of melamine nephrolithiasis in humans and animals [14, 20, 21]. Signs of lymphoplasmacytic or granulomatous tubulointerstitial inflammation and fibrosis can be observed in severe cases with larger crystals [13]. Ultrasonographic examinations indicate that these stones have a brown exterior with a colorless white core [22, 23].

## 3 MELAMINE TOXICITY

### 3.1 Prenatal Melamine Exposure (PME) and Gross Effects on the Brain

Melamine, a small, polar, water-soluble compound, can penetrate the placental barrier and reach the embryonic bloodstream. Melamine can bind to lipids, forming insoluble crystals such as melamine cyanurate when exposed to heat or alkaline conditions, resulting in adverse health effects such as the formation of kidney stones [24]. Animal models have demonstrated that maternal exposure to melamine causes it to accumulate in breast milk and then be transferred to the fetus' serum, amniotic fluid, and tissues, including the brain, kidneys, lungs, and heart [5]. It is revealed that melamine accumulation in brain regions such as the hippocampus causes a decrease in the ratio of hippocampal to brain weight. That loss of weight has been linked to cognitive dysfunction [25]. This exposure can disrupt calcium homeostasis and alter the hippocampus's histology, inducing apoptosis [6]. On top of that, activating oxidative stress at this region causes structural changes such as neural loss and necrotic neurons; these phenomena alter normal cell metabolism, for instance, by reducing membrane fluidity, disrupting cell barrier function, and ceasing cell membrane transporters; these factors are associated with cell death [16]. Melamine exposure inhibits perinatal neuronal excitability in the CA1 region of the hippocampus. There is a close relationship between glutamate receptors and the mechanisms that modulate synaptic plasticity. Both frequency and amplitude of Spontaneous excitatory postsynaptic currents (sEPSCs), which triggered numerous signal transmissions, were decreased in PME future generations [17]. According to El Hassar *et al*. (2007), alterations in the function of excitatory synapses lead to deficits in specific memory domains, such as spatial cognition [26].

In the Morris water maze (MWM) test, a well-known Learning and re-acquisition learning test, PME rodents demonstrated a significant impairment of spatial learning and memory. They could not adjust their conduct in response to a condition change [17]. As stated beforehand, a significant correlation exists between maternal melamine ingesting and congenital disabilities. Lei *et al*. (2019) state that PME does not affect adolescents' spatial learning ability. PME impairs cognitive performance in adolescent female rodents, but its age-dependent effects fluctuate. Adult and adolescent female rodents treated with PME exhibited impaired memory re-acquisition and decreased LTD in response to low-frequency stimulation. The impact of PME on LTP and NMDAR subunits varied significantly between adolescent and adult rodents, indicating that the effects are age-dependent. The study concludes that PME and hippocampal LTD do not independently regulate synaptic efficiency and spatial cognition but rather as a single entity. The anomalous adaptability of cognitive function may be due to the adverse effects of PME on long-term synaptic plasticity [17]. In adults, insoluble metabolites related to melamine are removed from cells, whereas in neonates, this occurs less frequently [14]. Consequently, melamine may exert age-related effects. Previous research indicated that the adverse impact of PME on the function of the hippocampus may persist into maturity. The mechanism underlying the prolonged impact of PME remains to be investigated. In addition, Tayem *et al*., 2019 established that PME promotes an increase in incomplete or absent bones, fewer ossified centers in the sternum and metacarpal bones, and no aberrant ossification in the metatarsal, cranium, pubic, or rib bones. In group 2, there was a significant increase in vertebral centers with absent or delayed bones and abnormalities in the thoracic and sacral centers. PME elicited dose-dependent bone ossification retardation [27].

### 3.2 Effects of Melamine on Long Term Potentiation (LTP) and Long Term Depression (LTD)

**Fig. (1):**
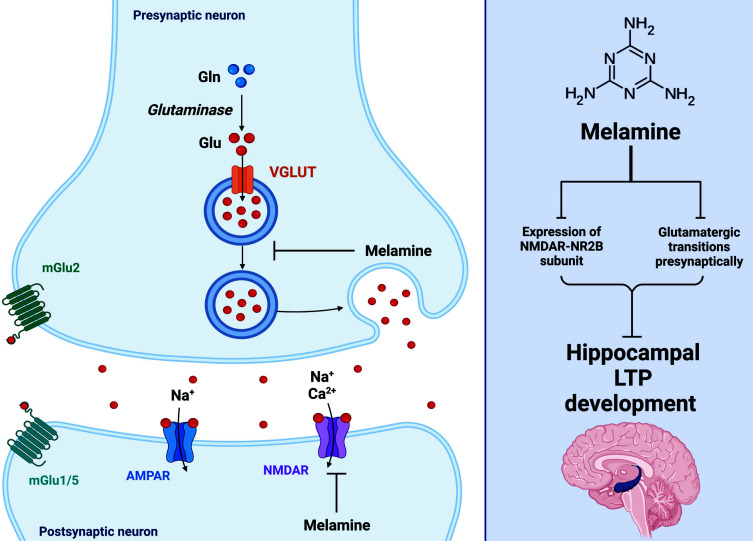
Long-term potentiation and the effects of melamine. Glutamate is widely regarded as a crucial element in the induction of LTP, which is known to rely on excitatory synaptic transitions. Melamine was shown to affect presynaptic glutamatergic transmission. Also, Melamine inhibits LTP by lowering the expression of the NMDAR-NR2B subunit. (Created with BioRender.com).

The hippocampus is a vital structure located deep within the brain's medial temporal lobe, which serves a crucial role in various cognitive processes, particularly learning and memory. The dentate gyrus is the entry location for information getting into the hippocampus [28]. It is essential for pattern separation, distinguishing identical experiences, and forming distinct memory representations. CA1 (Cornu Ammonis 1) is a crucial subregion for consolidating and retrieving long-term memories. It receives input from CA3 and transmits output to other brain regions. Memory impairments can result from CA1 impairments. CA3 (Cornu Ammonis 3) is involved in pattern completion and is responsible for forming and maintaining associations between various memory elements. It also contributes to quickly encoding and retrieving information [28]. Also, Hippocampal LTP, particularly at Schaffer collaterals (SC) to CA1 regions, is a model of learning and memory at the cellular level [20]. Neural activity patterns impact synaptic plasticity, a phenomenon underlying learning and memory. It is well-established that LTP development depends on excitatory synaptic transitions, and glutamate is regarded as an essential variable in the induction of LTP [29]. Biochemical manipulations can negatively impact synaptic plasticity, and excessive glutamate levels can impair brain function. Limiting the spillover of glutamate, glutamate transporters prevent the excessive activation of extrasynaptically located receptors, which can impair synaptic plasticity. Melamine could interact with presynaptic glutamate receptors [29]. These receptors, including metabotropic glutamate receptors (mGluRs), can modulate the release of neurotransmitters. Melamine could affect the presynaptic regulation of glutamatergic transmission by altering the function of these receptors [29]. Therefore, it has been hypothesized that melamine's effect on excitatory synaptic transmissions is one of the mechanisms by which plasticity is altered [10]. Even though melamine can disrupt presynaptic calcium homeostasis, its primary effect on LTP and LTD is a decrease in the expression of the GluN2B subunit of NR2B in both adolescent and adult rodents. In contrast, only adolescent rodents exhibited a reduction in GluN1B subunits (Fig. **1**), demonstrating that the effects of melamine vary with age. Consistent with these findings, Yang *et al*. (2012) reported that administration of melamine to hippocampal slices of postnatal 10-14-day rats decreased synaptic transmissions in the SC to CA1 pathway and that young rats were more affected than adult rats because their synapses underwent extensive remodeling. Changes in the location and composition of glutamate receptors in the hippocampus may also contribute [15]. This decrease in receptor expression is linked to plasticity and memory deficits. Thus, melamine dramatically inhibits hippocampal CA1 NMDAR-dependent LTP and LTD induction [30]. In addition, the abnormally enhanced Paired-pulse facilitation (PPF) ratio revealed this reduction [15]. Nichollas *et al*. (2008) observed in transgenic rats that the function of the Ser/Thr protein phosphatase 2A was inhibited in the forebrain, NMDAR-LTD was diminished, and reversal learning in the water maze was disrupted [31]. Similarly, Zeng *et al*. (2001) found that mutant rodents with reduced SC-CA1 LTD exhibited deficiencies in habituation to a new environment [32]. Melamine inhibits LTD in adolescent and adult rats [17] by preventing the nervous system from acquiring further information or increasing the interference between new learning and previously acquired information.

These studies indicate that LTD, like LTP, is crucial in behavioral flexibility [6]. Which rodents under melamine lost it.

### 3.3 Impairment of Cognitive Function

The examination of the activity of a single neuron in behavioral and mental disorders relies heavily on the synchronized oscillations of biological rhythms. According to studies, this synchronization between CA3 and CA1 subregions of the hippocampus underlies cognitive behaviors. A specific neuronal oscillation in one particular cognitive behavior demonstrates that this synchronization is diminished in mental disorders [33]. In addition, Colgin *et al*. (2009) confirmed that low and high gamma oscillations in the hippocampus contribute independently to physiological processes [34]. In line with the findings of Xu *et al*. (2013), the theta phase in the CA3 region modulates the low gamma amplitude rather than the high gamma in the CA1 region; these theta-low gamma waves disappeared in melamine-treated groups, indicating that this neural network is weakened [33]. Low gamma rhythm has been reported to be implicated in memory retrieval [35]. It has been demonstrated that glutamatergic transmission in the hippocampus is a critical factor in the synchronization of theta waves and that this synchronization is closely related to hippocampal LTP [33]. NMDA receptors modulate synaptic currents in -amino-3-hydroxy-5-methyl-4-isoxazolepropionic acid (AMPA) receptors; consequently, it appears that the effects of melamine on evoked postsynaptic currents (eEPSC) are predominantly mediated by NMDA receptors. Since it has been demonstrated that NMDA receptor inhibitors such as MK-801 do not inhibit eEPSC, melamine functions *via* presynaptic mechanisms. Total prenatal and postnatal exposure to melamine impairs cognitive performance [17]. Several melamine-treated animal models exhibit hippocampal neuron loss and early synaptic decay, both associated with cognitive impairment [9]. The aforementioned changes in the developing hippocampus of a fetus result in irreversible neuronal changes and, consequently, impaired memory and learning, which are believed to be encoded by alterations in synaptic strength [36]. The MWM test revealed a significant prolongation of escape latency in the IT stage in the melamine-treated group, impairing their learning capacity. The rats were unable to use their reference memory during the SET stage, and they ultimately performed inadequately during the RT stage. They could not devise a new strategy in a new environment. The ability to swiftly switch from the previous objective to the new target has been compromised. These findings suggest that melamine not only inhibits previously learned strategies but also precludes the formation of new strategies in novel situations [31].

Performance in reversal MWM tasks depends on cognitive flexibility; to achieve this, the subject must simultaneously discard the previously acquired strategy and create a new one. Consequently, based on behavioral evaluations, PME may impair the ability to learn [6]. Consistent with these findings, another study demonstrated that in melamine-exposed rodents, the amount of time spent in the target quadrant and the number of crossings in the target quadrant decreased significantly, indicating impaired memory function [33]. It should be noted that An *et al*. (2011) found that melamine did not appear to influence motor activity [20] because melamine-treated groups maintained the same swimming speed as the control group. In addition, protracted melamine exposure of 400 mg/kg/day (approximately ten times the human tolerable daily consumption) for 35 days disrupts the cholinergic system of the hippocampus in rodents. It may be due to acetylcholinesterase hyperactivity; as a consequence, acetylcholine levels decline, which appears to be associated with memory and learning impairments [6, 31]. Finally, it can be concluded that prenatal and postnatal melamine exposure impairs memory and learning on the MWM test [9]. A separate study revealed that the melamine-exposed groups had significantly reduced acetylcholine activity than the control group. Nonetheless, the results of some experimental studies disprove that melamine and cyanuric acid must be present for damage to occur and imply that even minimal melamine exposure may pose health risks to nerves, contrary to the belief that damage is limited to the kidneys [14]. Recent research has demonstrated that melamine impacts the induction of long-term depression, which is associated with novelty acquisition and memory consolidation, despite earlier findings that melamine has neurotoxic effects on hippocampus-dependent learning and reverse learning. A low (200 mM/L) or high (400 mM/L) dose of melamine was injected 30 minutes prior to testing to determine if behavioral performance was dose-dependent. On the Y-maze reversal learning criterion, both the low-dose and high-dose groups of rodents outperformed the control group. The proportion of trials in which the low-dose group met the criterion was significantly lower than the high-dose group. It was determined that there were no significant differences in running pace, which is consistent with previous investigations. Moreover, the rodents' motivation levels did not fluctuate during the press lever test, ruling out this hypothesis as the cause of the impairment [10].

Unknown was whether melamine altered the capacity to learn and the strategy employed when exploring the baited area. Consequently, rodents were trained in a cross-maze task and then tested with a probe test 30 minutes after receiving a melamine infusion. None of the melamine-treated rats displayed a preference for one strategy over another, but the control animals did. Compared to the low-dose group, the proportion of rodents using the spatial strategy decreased dramatically in the high-dose group. Our findings suggest that melamine inhibits using a spatial strategy, resulting in the contrary impairment in the place-related task [37].

Brain-Derived Neurotrophic Factor (BDNF) is a protein that promotes the formation and strengthening of synapses between neurons, which is necessary for learning, memory, and synaptic plasticity. BDNF is crucial during brain development but is also involved in various functions throughout life. Evidence shows that spatial training can increase BDNF expression [38] and initiate BDNF signaling in the hippocampus. It has been discovered that melamine has neurotoxic effects on hippocampus-dependent learning and reverse learning. In a study involving rodents, intra-hippocampal melamine injection led to a dose-dependent increase in reversal learning trials to the criterion without locomotion or motivation defects. The learning-induced BDNF level in HPC neurons was substantially decreased, and bilateral intra-hippocampal BDNF infusion effectively counteracted the inhibiting effects of melamine on neural correlates of reversal performance. These results indicate that melamine infusion into the hippocampus inhibits training-induced BDNF expression without affecting basal BDNF expression [37].

## 4 EFFECTS OF MELAMINE ON ION CHANNELS

Numerous toxins and pharmaceuticals target the membrane channels of the cell. Melamine appears to be neurotoxic to hippocampal cells by interfering with calcium homeostasis. As a second messenger, calcium is essential for biological processes, including regulating cell excitability, cell transmission, and synaptic plasticity. Toxin-induced cell mortality increases intracellular calcium concentration or impedes the function of its transporters [14]. Apoptosis is associated with an abnormal increase in intracellular calcium levels, which may occur in three stages. In the initial phase, there is an increase in calcium levels, which serves as an initial signal. The average cytosolic calcium concentration may be below normal in the second or moderate stage. In the third stage or execution phase, however, a significant increase in intracellular calcium levels is observed. Proteases and endonucleases are activated, resulting in the formation of apoptosomes [39]. In addition to the hippocampus, such reactions have also been observed in PC12 cell lines [2].

Given the relationship between calcium and potassium currents, melamine also alters potassium currents. Potassium channels are crucial in modulating neuronal excitability, division, differentiation, and mortality [40]. It is well-known that a decrease in potassium currents increases the excitability of neurons. Changes in neuronal excitability are regulated and modulated by dendritic transient potassium channels [20] following induction of long-term potentiation. There are two primary varieties of potassium currents: They can inhibit both voltage-dependent transient outward K+ currents (IA) and delayed rectifier K+ currents (IK), with an inhibitory effect on IA occurring before IK. However, its inhibitory effects on IK are more potent than on IA. It was observed that melamine substantially shifted the activation curve of IK to negative potentials but had no effect on IA activation neurons. Furthermore, a substantial negative shift in the steady-state inactivation of IA was only observed at large melamine concentrations [40]. A distinct change in the flow of these channels results in neuronal dysfunction. Schroder *et al*. (2000) demonstrated that decreased IK current would result in neuronal hyperexcitability and epilepsy [41].

**Fig. (2):**
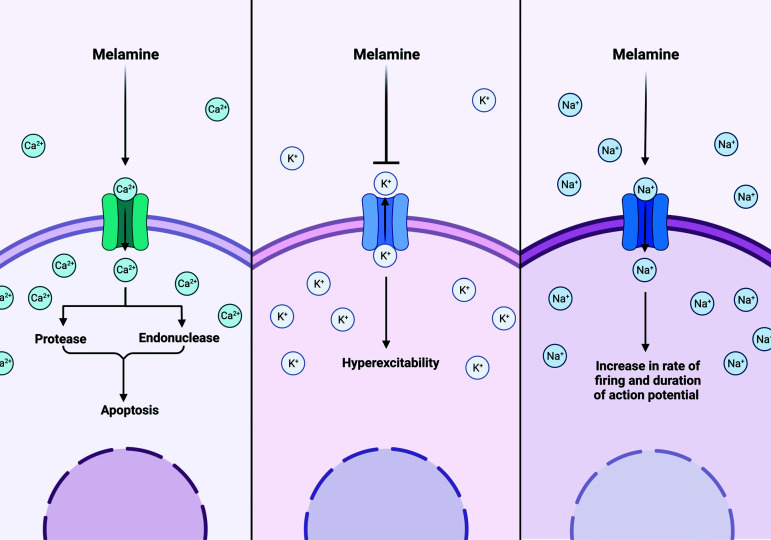
Melamine and ionic currents. Melamine is cytotoxic to the hippocampus because it disrupts calcium homeostasis. Toxins either increase calcium levels in cells or disrupt the transporters that carry calcium across the body. The calcium level in the cells dramatically increases. Proteases and endonucleases trigger apoptosomes. Also, neuronal excitability is reduced when potassium currents are blocked and identified Epilepsy as a result of Excessive Excitability in Neurons. On the other hand, action potentials in hippocampus neurons are generated and transmitted *via* voltage-gated sodium channels (VGSCs), which melamine disrupts. These modifications smooth down the sodium channel's inactive-to-active transition. As a result, the action potential and firing rate increase. (Created with BioRender.com).

Voltage-gated sodium channels (VGSCs), which are responsible for initiating and propagating action potentials along the axons of hippocampal neurons, are also affected by melamine exposure. According to electrophysiological investigations, melamine exposure modifies these channels' parameters. It alters the action potential characteristics and repetitive firing patterns by inducing a negative shift in the activation and inactivation curves. In this case, sodium channels cannot recover from a state of inactivity. Consequently, in the presence of melamine, there is a maximal reduction in sodium current [42]. It appears that melamine binds to a receptor site in the S3-S4 loop at the extracellular end of the S4 segment in domain II of the -subunit and alters the channel's structure through covalent or electrostatic connections [42]. Only at high concentrations of melamine (about 500 g/ml) are these effects observed, and at low concentrations (5 g/ml), melamine has a low affinity for sodium channels. Yan *et al*. (2010) discovered that at comparatively high concentrations of melamine, the peak amplitude, overshoot, and voltage threshold of the evoked single action potential were significantly reduced [42]. Such alterations indicate that sodium channels transition from an inactive to an active state more readily, allowing the voltage sensor portion of these channels to move more freely outward—consequently, the discharge rate and action potential duration increase (Fig. **2**).

## 5 MELAMINE-INDUCED OXIDATIVE STRESS

Although Mast *et al*. (1983) found that approximately 90% of melamine entering the body is excreted through the kidneys within 24 hours [43], Yang *et al*. (2011) demonstrated that elevated melamine concentrations lead to its accumulation [10]. Several factors contribute to melamine's entry into the brain parenchyma, though the precise mechanisms of its accumulation in the brain are not entirely understood. Passive diffusion can transport melamine across the Brain-Blood Barrier (BBB), particularly at high concentrations [44]. Additionally, studies suggest that organic cation transporters (OCTs) expressed on the blood-brain barrier (BBB) can absorb melamine. These transporters make it possible for melamine to enter the brain [44]. Importantly, melamine toxicity and accumulation in the brain have been investigated predominantly in the context of acute poisoning or high-dose exposures. Chronic exposure to low doses of melamine may have distinct effects, requiring further study. Various mechanisms can lead to melamine accumulation in the Cerebral Cortex, Basal Ganglia, Hippocampus, Cerebellum, and Ventricles.

When there is an imbalance between the production of reactive oxygen species (ROS) and the body's capacity to detoxify or restore the damage, oxidative stress occurs. ROS are oxygen-containing, chemically reactive molecules produced as byproducts of cellular metabolism. Excessive ROS production can disrupt cellular components, resulting in various pathological conditions, such as neurological diseases. Extensive research has found that melamine induces oxidative stress [16, 45, 46], which damages macromolecules such as lipids, proteins, and nucleic acids [47]. When oxidative stress occurs, nuclear respiratory factor 2 (Nrf-2), mitogen-activated protein kinase (MAPK), and nuclear factor-jB (NF-jB) turn active and, in conjunction with a change in the mitochondrial permeability transition pore, damage the inner membrane of mitochondria and organelles [12]. Melamine's toxicity contributes to the disruption of redox balance and oxidation-antioxidation homeostasis. BDexposure induces oxidative stress in hippocampal neurons due to the aberrant production of reactive oxygen species (ROS) and apoptosis [48]. Expanding levels of reactive oxygen species decrease endogenous antioxidants while increasing superoxide anion and hydroxyl radicals [16].

Malondialdehyde (MDA) and Superoxide Dismutase (SOD) induce lipid peroxidation by attacking the cell membrane, according to Ehninger *et al*. Melamine, by increasing the level of superoxide anion, hydrogen peroxide, and hydroxyl radical, directly or indirectly alters cell signaling pathways, including Nuclear factor kappa B (NF-κB) pathway, Mitogen-Activated Protein Kinase (MAPK) pathways, Phosphatidylinositol 3-kinase (PI3K)/Akt pathway, Calcium signaling pathway, and Bcl-2 family proteins and mitochondria-mediated apoptosis, which eventually leads to cell death [2]. Caspases, one of the activated proteases in cell death, are responsible for chromatin condensation and DNA fragmentation [14]. Caspase-3 is frequently required for morphological changes such as neuronal loss, diminished nucleus, decreased cytoplasm [2, 47] and ATP levels, and increased lactate, which decreases neuronal pH and causes cell death. This neuronal loss reduces the average weight of the hippocampus, as observed in groups treated with melamine [9].

**Fig. (3):**
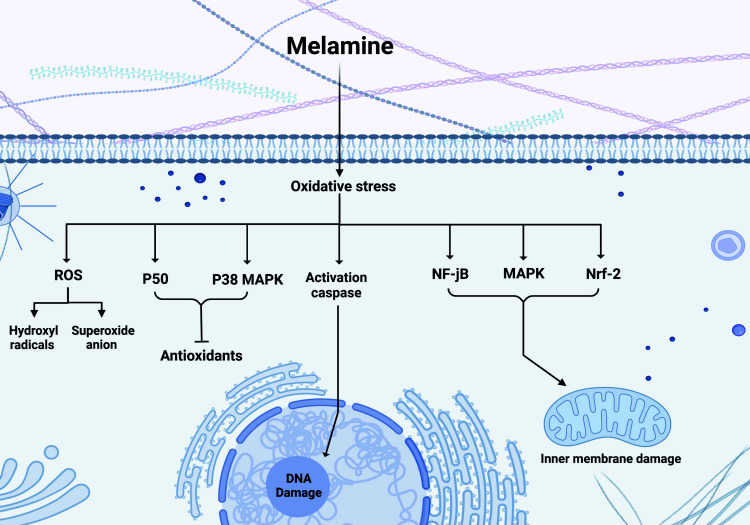
Melamine causes oxidative stress. Melamine is regarded as a well-known stress-inducing agent. Damage to the inner membrane of mitochondria and other organelles is caused by the activation of Nrf-2, MAPK, and NF-jB in response to oxidative stress. When ROS levels rise, superoxide anion and hydroxyl radical production also increase. Also, caspase is an active protease that induces chromatin condensation and DNA fragmentation during cell death. In addition, oxidative stress-related damage activates the p38 MAPK pathway and p50 activity, suppressing the antioxidant effect. (Created with BioRender.com).

Nitric oxide (NO) derived from mitochondrial NOS can also modulate mitochondrial ROS production. NO can be converted into reactive nitrogen species, including nitroxyl anion and peroxynitrite. By generating peroxynitrite, its high concentration can react with superoxide anion to cause DNA damage and irreversible alterations in proteins, such as tyrosine nitration or thiol oxidation [47]. Moreover, oxidative stress impairments activate the p38 MAPK pathway [49] and p50 activity; both circumstances inhibit the antioxidant effect [50]. Using an NADPH oxidases (NOX) inhibitor decreased the production of reactive oxygen species (ROS) induced by melamine, indicating that activation of the NOX pathway is essential for ROS activation [50]. NOX2-3-4 is highly expressed in the renal vascular system [51] and is essential for developing kidney disorders. Specifically, macrophages and kidney cells express NOX2 [50]. Melamine toxicity in the kidney can be induced by activating these pathways (Fig. **3**).

Evidence shows that cells use various methods to reduce the damage caused by oxidative stress, such as repairing the defect or utilizing different antioxidants [2]. In the mitochondrial matrix, Superoxide dismutase (SOD) is the first line of defense against reactive oxygen species (ROS), converting it to hydrogen peroxide before being metabolized by KAT and Glutathione peroxidase (GSH-px). By measuring oxidative stress markers such as MAD, the role of oxidative stress in developing cognitive disorders can be examined [47].

In the intricate realm where melamine-induced oxidative stress converges with the guardianship of vitagenes, vitamins C and E emerge as pivotal players, weaving a narrative of cellular resilience and defense. Melamine, known for its capacity to disturb the delicate equilibrium of ROS and induce oxidative stress, sets the stage for the protective prowess of vitamins C and E. These antioxidants, highlighted in both texts, stand as beacons of hope in countering the detrimental effects of melamine on cellular components such as lipids, proteins, and nucleic acids. The vitagenes network, comprising heat shock proteins (Hsps) and heme oxygenase-1, intricately intersects with the antioxidant properties of vitamins C and E. As the melamine narrative unfolds, the importance of maintaining redox balance and oxidation-antioxidant homeostasis becomes evident. Vitamins C and E, recognized for their ability to activate SOD, CAT, and GSH-px, offer a formidable defense against oxidative stress markers such as MDA [2, 52, 53]. This duo of vitamins not only engages in the frontline defense against ROS but also holds promise in the modulation of cellular signaling pathways, including NF-κB, MAPK, and apoptosis-related pathways. In the intricate dance of molecular interactions, vitamins C and E showcase their potential to reduce neuronal injury and cognitive impairment caused by melamine-induced oxidative stress, ultimately impacting synaptic plasticity and cognitive function [47].

ROS or NO regulates its activity and expression, as well as mechanisms like post-translational modifications or the ubiquitin-proteasome pathway [54]. NO alters the activity of intracellular signaling proteins such as NMDA receptor, protein kinase C, activating protein-1, Ras, and caspase-3. NO plays a pivotal role in neuroprotection through diverse mechanisms, complementing its modulation of intracellular signaling proteins. Experimental models illuminate the multifaceted neuroprotective effects of NO. In primary rat cerebellar granule cells, inhibition of NO synthesis increased apoptotic cell death *via* caspase-3 activation, a response mimicked by the sGC inhibitor ODQ and reversed by NO donors or cGMP analogues. This protective pathway involves Akt and CREB, crucial in neurotrophin-mediated survival and defense against neurodegenerative challenges [55]. In the NMDA-mediated neurotoxicity model, NO shields against excitotoxic cell death by S-nitrosylating NR1 and NR2 subunits, mitigating intracellular Ca^2+^ influx. NO's cytoprotective role extends to inhibiting caspase activity through S-nitrosylation, reducing caspase 3 and caspase 9 activation in cortical neurons [56-58]. Surprisingly, this inhibition doesn't correlate with increased neuronal viability, suggesting alternative, caspase-independent forms of cell death [58]. Moreover, NO induces haem oxygenase 1, an early responder to oxidative stress. In the brain, NO triggers haem oxygenase 1 in rat astrocytes, microglia, and the hippocampus. The upregulation of haem oxygenase 1 and subsequent elevation of biliverdin, transformed into the antioxidant bilirubin, constitute a secondary neuroprotective mechanism under pro-oxidant conditions [59, 60]. NO's neuroprotective prowess manifests through intricate signaling pathways involving Akt, CREB, and caspases. S-nitrosylation emerges as a key molecular mediator, mitigating excitotoxicity and inhibiting caspase activity. Additionally, haem oxygenase induction contributes to the antioxidant defense. NO's ability to navigate this intricate network highlights its significance in safeguarding neurons against various threats, showcasing a nuanced interplay of molecular mechanisms in neuroprotection [61].

Melamine-induced oxidative stress appears to be mediated by the activation of these pathways, so it would appear that antioxidant therapy is an appropriate method for mitigating these effects. Table **1** provides a comprehensive summary of numerous melamine impact studies [62-70].

## 6 AUTOPHAGY REVERSES THE EFFECTS OF MELAMINE

Autophagy is an oxidative stress-activated cell defense mechanism that recycles intracellular organelles [66]. Autophagosomes engulf proteins and intracellular organelles and are then transferred to lysosomes for digestion. Previous research has demonstrated that autophagy is an effective method for mitigating the side effects of melamine [71]. Similarly, autophagic activating pharmaceuticals such as Rapamycin can restore cognitive function and synaptic plasticity in melamine-treated rodents' hippocampal neurons [72].

Similarly, neuronal recording experiments conducted by Fu *et al*., 2016 revealed an increase in the slope of field excitatory postsynaptic potential (fEPSP) in rats treated with rapamycin. A rise in these gradients indicates enhanced synaptic transmissions and enhanced memory and learning. It is well-known that GluN2B subunit-containing NMDA receptor synaptophysin and postsynaptic density protein 95 (PSD-95) as pre and post-synaptic structural proteins are intimately associated with synaptic plasticity and cognitive process in the hippocampus. It has been observed that melamine decreases the expression levels of both NR2B and PSD-95, but rapamycin administration reverses these effects [48]. It also substantially increased the expression of LC3-II [70], Beclin-1 [48], and PSD-95 and downregulated the mechanistic target of Rapamycin (mTOR) signaling, which improved melamine-induced impairments by activating autophagy pathways [73]. Autophagy induced by rapamycin appears to be an effective therapeutic mechanism for neurological diseases.

PC12 cells are a type of catecholamine cells that synthesise, store and release norepinephrine and dopamine. Melamine inhibits the proliferation of PC12 cells in a dose- and time-dependent manner [72], in addition to the hippocampus. Activating autophagy in PC12 cell lines can protect them from H_2_O_2_ production-induced injury [48]. The autophagy-related (Atg) genes, including MAP1LC3B (encodes LC3), Atg7, and BECN1 (encodes beclin-1), are required for autophagy induction [74-76]. LC3 (microtubule-associated protein 1A/1B-light chain 3) is crucial in autophagy. There are two forms of LC3: LC3I (cytosolic form) and LC3II (lipidated form). Conversion of LC3I to LC3II is a crucial autophagy activation marker. During autophagy, LC3I is known to cleave and transform into LC3II [77]. Consistent with this, Wang *et al*., 2014 demonstrated that the level of autophagosomes increased markedly after 36 hours of melamine exposure. In the Western blot assay, they also observed the conversion of the soluble form of LC3I to the lipid form of LC3II, indicating the activation of autophagy [72]. By facilitating the conversion of LC3I to LC3II, Atg7 plays a critical role in the initiation and progression of autophagy. It also activates Atg12, which combines with Atg5 to create a complex essential for autophagy. Involved in the elongation of the autophagosomal membrane and subsequent formation of autophagosomes is the Atg12-Atg5 complex. Atg7 can increase LC3 homologs and the ratio of LC3I to LC3II. The formation of targeted, complex autophagosome vesicles indicates the activation of autophagy-related pathways [78]. Other signaling pathways, including Wnt signaling and the p38-NF-B pathway, may modulate autophagy mechanisms effectively [72].

## CONCLUSION

Melamine is present everywhere, making it hard to avoid consuming it. Melamine, also known as 2,4,6-tri amino-s-triazine, is a nitrogen-rich chemical compound that creates formaldehyde resins. Many different products and processes make use of this molecule. Melamine products include laminates, adhesives, plywood, flooring, paint pigments, and even furniture. There are several entry points for melamine into the human body. For example, melamine and formaldehyde are absorbed into the body by ingestion after being released from melamine food containers due to extended exposure to microwave heat or the use of acidic foods.

The most common clinical symptoms of melamine toxicity in humans and animals are kidney and bladder stones, which are caused by crystal formation in the urine ducts, as well as hematuria, crystalluria, urolithiasis, nephrolithiasis, epithelial hyperplasia, and melamine toxicity.

Melamine has a molecular size that makes it absorbable *via* the gastrointestinal system and potentially crosses the placental barrier. Animal studies have shown that melamine accumulates in breast milk and is absorbed by the fetus *via* the mother's blood and amniotic fluid when exposed to the chemical during pregnancy. When melamine is absorbed, it traverses the blood-brain barrier (BBB) and exhibits an adverse effect on the nervous system. Cognitive impairment has been linked to melamine accumulation in specific brain regions, including the hippocampus. As a direct result of these changes in the embryonic hippocampus, memory and learning are impaired, as these functions are thought to be stored through the strength of synapses.

Calcium, potassium, and sodium balance seem to be disturbed in melamine-toxic hippocampal cells. Activating autophagy may be all that's needed to lessen melamine's side effects. Autophagy-activating drugs like Rapamycin may also help restore normal cognitive function and synaptic plasticity in hippocampus neurons in melamine-treated rats.

Nonetheless, much more effort is required to reestablish public trust in melamine-free food. Melamine is widely used as an adulterant worldwide, posing significant health risks. Consequently, there is an urgent need to improve our understanding of the pathobiology of melamine exposure to develop therapeutic options in the coming days. In conclusion, we agree that encouraging people to eat food that hasn't been tainted with melamine should be a top priority since it provides an essential means of protecting human health and mitigating the dangers of melamine exposure.

## Figures and Tables

**Table 1:** An overview of melamine-induced toxicity.

**-**	**Model**	**Time of Exposure**	**Age/Gender**	**The Way of Exposure**	**Dose of Melamine**	**Results**	**References**
**Prenatal melamine exposure (PME)**	Wistar rats	Four weeks	3-week-old Male	Oral administration (intragastric)	300 mg/kg/day	Neural loss and morphological change in CA1 area of the hippocampus, striatum, cortex, and amygdala - reduced ATP content - impaired memory and learning	[16]
	Wistar rats	Gestational period	10-week-old Female	Gavage	400 mg/kg/day	Disturbed re-acquisition ability - age-dependent effects - Cognitive deficits in adolescents and adults - change in synaptic function	[17]
	Children	_	1 to 96 months Female/male	_	Melamine content: Patients with liver lesions 1331.3 ± 1207.3 (mg/kg) Patients with liver lesions 1295.3 ± 967.1	Urinary stones- liver abnormalities	[21]
	Rats	6^th^ to 20^th^ day of pregnancy	Adult female	Gavage	300 or 400 mg/ kg/day, respectively.	Reduction in ossified centers of offspring- ossification abnormalities	[27]
	Wistar rats	Gestational period	10-week-old Female/male	Gavage	400 mg/kg/day	Dysfunction of the hippocampus - reference memory and spatial learning deficits - cognitive functions impairments - impairments of LTP	[36]
	Sprague-Dawley rats	Gestational day 17/ postnatal period	Postnatal day 14	Gavage	Maternal exposure: ∼6-7 mgneonatal rats: (∼0.3-0.6 mg)	Melamine can cross the placenta and reach the amniotic fluid and breast milk - umbilical circulation can remove melamine from the fetus - in plasma, it has 3 hours half-life and then can be excreted in urine - it can accumulate in the kidney	[62]
	Rats	Gestation day (GD) 13 to GD 20 to	10 to 12 week-old Female/male	Gavage	40 and 400 mg/kg	Dose-dependent effects of melamine - melamine cause an elevation in plasma uric acid, plasma creatinine, and blood urea nitrogen concentrations	[63]
**Long-term potentiation and depression**	Wistar rats	Gestational period	10-week-old Female	Gavage	40 mg/mL	Learning and memory deficits - LTD/LTP were reduced	[6]
	Wistar rats	Four weeks	3-week-old Male	Oral	300 mg/kg/day	Gaining body weight in melamine-treated groups was lower - impaired spatial learning ability - disturbance in spatial memory performance - decreased fEPSP slopes so attenuated synaptic plasticity in CA1 region - frequency and not the amplitude of mEPSC was reduced - affecting glutamate release and depressed both Eepsc and m EPSC - pre and not postsynaptic change in glutamate transmission	[10]
	Sprague-Dawley rats	-	10-14 day-old 21-28 day-old 2-3 month-old Male	Oral administration	5 and 25 mg/kg	Synaptic transmission in the Shaffer-collateral pathway was depressed in a dose and age-dependent manner - because of synaptic remodeling, CNS of young rats is more affected than old rats - considerably decreased hippocampal LTP and LTD	[15]
	Wistar rats	Four weeks	3-week-old Male	Gavage	300 mg/kg/day	Haematuria and piloerection and decreased spontaneous activity- abnormal melamine concentration in the hippocampus - decrease in body weight - effects on physical growth - disruption of spatial memory and learning - reduced LTP in the CA1	[20]
	Rats	-	-	Intraperitoneal injection (i.p.) and intrahippocampal (i.h.)	20 mg/kg i.p. 200 mM/µL i.h.	Impairment of spatial memory - disruption of consolidation memory without affecting acquisition and retrieval memory - reduced NR2B and NR1 subunits of NMDAR - suppress hippocampal LTP/LTD dependent NMDA receptor subunit	[30]
	Wistar rats	Four weeks	4-week-old Male	Gavage	300 mg/kg/day	Neurotoxic effects of melamine on LTD - decreasing LTP from Schaffer collaterals to hippocampal CA1 region - abnormal levels of Ach/AChE in the hippocampus - Suppression of previous and new behavioral strategies	[31]
	Sprague-Dawley rats	Four weeks	3-week-old Male	Gavage	300 mg/kg/day	Melamine accumulation in the hippocampus - reduced neural information flow in CA3-CA1 pathway - disruption of the expression of AChE and ACh in the hippocampus	[64]
	Wistar rats	4-weeks	3-week-old Male	Gavage	300 mg/kg/day	Long-term synaptic plasticity in the hippocampus was reduced - the ability of reversal learning was disrupted - Vitamin therapy reduced the effects of melamine on cognitive function	[65]
**Impairment of Cognitive Function**	Wistar rats	Four weeks	3-week-old Male	Oral administration	300 mg/kg/day	CA3-CA1 synaptic plasticity, as well as cognitive function, was disrupted - theta and gamma rhythms and frequencies were decreased - information flow in the hippocampal CA3-CA1 pathway was suppressed	[33]
	Long Evans rats	-	Male	-	-	Change in low and fast gamma oscillations and information flow in the hippocampus	[34]
	Wistar rats	Four weeks	10-week-old Female	Gavage	400 mg/kg/day	Dysfunction in CNS - Young animals were most affected - reference memory and learning impairments - disrupted cognitive functions - presynaptic excitatory neurotransmission and LTP suppressed	[36]
	Sprague-Dawley rats	-	Male	intrahippocampal (i.h.)	200 mM/μL or 400 mM/μL	Disrupted cognitive flexibility- Learning disabilities induced by melamine reverse by intra-hippocampal application of BDNF reduce the firing rate of pyramidal neurons and inhibits BDNF-mediated neural activity	[37]
-	Wistar rats	-	3-week-old Male (hippocampal slices)	Perfusion system	5 ×10^-4^, 5×10^-5^, and 5×10^-6^ g/mL	The amplitude of eEPSCs in the CA3-CA1 pathway was increased - Melamine effects on eEPSCs were Independent of the NMDAR - paired-pulse ratio (PPR) was reduced - melamine-induced autophagy in the CA3 pyramidal cells	[66]
	Wistar rats	Postnatal day ~ 53	3-week-old Male	Gavage	300 mg/kg/day	The flow of information in the path CA1 to CA2 decreased - cognitive dysfunction -melamine changes the function of the GluR2/3 subunit of AMPA receptors in the CA1 region	[67]
**Effects of melamine on ionic currents**	Rats	-	-	PC12 cells, derived from the adrenal medulla	990 µg/mL	Cell viability is decreased by melamine and vitamin C/E, or their combination reverses it and has antioxidative activity -vitamins inhibit the activity of caspase-3 and attenuated apoptosis induced by melamine	[2]
	Wistar rats	Melamine exposure on the eighth day of culture	-	Hippocampus tissues culture	156, 312, and 625 mg/mL	Morphology and activity of caspase-3 in the hippocampus were changed - Insoluble metabolites caused by melamine cause cellular changes	[14]
	Wistar rats	-	10-14 days of ages Male	Hippocampal slices exposure to melamine	5×10^-4^, 5×10^-5^, 5×10^-6^ g/mL	Inhibited IK and IA, and this effect on IA was induced earlier - the blocking potency was stronger on IK than IA - the activation curves of IK shifted to a negative potential by melamine	[40]
	Rats	Slices were exposed to melamine for 5 min	-	Hippocampal Slices	5×10^-4^, 5×10^-5^, 5×10^-6^ g/mL	Melamine impaired electrophysiological properties of voltage-gated sodium channels (VGSCs), such as negative shifting of inactivation/ activation curve - a pattern of repetitive firing and action potential properties changed - at high concentrations of melamine, VGSCs properties were altered. As a result, overshoot, voltage threshold, and peak amplitude of single action potential were decreased	[68]
**Oxidative stress**	Wistar rats	Gestational period	10-week-old Male/Female	Gavage	400 mg/kg	Pre-postnatal melamine exposure considerably disrupted spatial memory, learning abilities, synaptic plasticity, and cognition - impaired cognitions - neuromotor disability - impaired reference memory - decayed LTP and memory dysfunction was more severe in prenatal melamine exposure groups than post-one	[9]
	Rats	Four weeks	3-week-old Male	Oral administration (intragastric)	300 mg/kg/day	elevated hydroxyl and superoxide onion free radicals and MAD levels in the hippocampus -indicating oxidative damage	[16]
	Wistar rats	Four weeks	3-week-old Male	Gavage	300 mg/kg/day	Learning and memory impairments induced by melamine-morphological changes, such as the shrunken nucleus, neuronal loss, and reduced cytoplasm,	[48]
	-	-	-	-	-	were seen in melamine-treated groups - vitamin therapy reverses these effects; they activated antioxidant defense system and leads to decline in lipid peroxidation and ROS levels - oxidative stress and depletion of endogenous antioxidants occurred in the melamine-treated hippocampus - MDA level, CAT, GSHPx and SOD, activities, was considerably restored after vitamin therapy - vitamins therapy regulated oxidation- antioxidation homeostasis	-
	Wistar rats	Four weeks	3-week-old Male	Gavage	300 mg/kg/day	Melamine-induced cognitive impairment was reduced by rapamycin - rapamycin also increased the levels of synaptic proteins and decreased the intracellular level of ROS - it caused up-regulation of autophagy in melamine exposure groups and improved cognitive functions - lower expressions of PSD-95 and NR2B proteins induced by melamine reverse by rapamycin - the level of decline-1 and the ratio of LC3-II/LC3-I were notably enhanced in the rapamycin-treated groups	[49]
	Murine macrophage and human embryonic kidney cell line	1 or 24 h	-	-	1 pM, 1 nM, and one l M	Melamine toxicity mediated by activation of NOX and NFκB-related pathways in macrophages- melamine increased depression of NFκB P65, IκBα, and DNA binding activities p50 - it also increased inflammatory factors such as PGE2, NOX, COX-2, in embryonic kidney cells macrophages	[50]
	Rats	20 min incubation	-	NRK-52e cell line	0, 8, 16, and 24 mM	Melamine activated the P38 MAPK pathway in the NRK-52e cells - it improved intracellular ROS levels and elevated necrotic and apoptotic percentages by activating the p38 MAPK signaling pathway of the NRK-52e cells dose-dependent manner	[51]
	Rats	12, 24, and 36 h incubation	-	PC12 cells, derived from the adrenal medulla	(0, 33, 330, 990, 1980, and 3300 µg/mL)	Inhibitory effects of melamine on the viability of PC12 cells - inducing oxidative stress and apoptosis - inhibited differentiation of PC12 cells	[69]
	Rats	36 h	-	PC12 cells, derived from the adrenal medulla	0, 99, 330, 990, 1980, and 3300 μg/mL	Melamine exposure caused activation of autophagy - the ratio of LC3II/LC3-I was increased - Autophagy, by inhibiting ROS generation, significantly reduced melamine-induced PC12 cell	[70]
	African catfish juveniles *C. gariepinus*	45days	-	Feeding	3.0 g Kg^-1^	Neurotoxicity and oxidative stress were induced in melamine-exposure fishes - Elevated levels of serum factors, such as ALT/ AST, indicated hepatic damage	[71]

## LIST OF ABBREVIATIONS

AMPA: α-amino-3-hydroxy-5-methyl-4-isoxazole Propionic Acid
Atg: Autophagy-related
BBB: Brain-blood Barrier
Bcl-2: B-cell Lymphoma 2
CNS: Central Nervous System
eEPSC: Evoked Postsynaptic Currents
fEPSP: Field Excitatory Postsynaptic Potential
GSH-px: Glutathione Peroxidase
Hsps: Heat Shock Proteins
IA: Voltage-dependent Transient Outward K^+^ Currents
IK: Delayed Rectifier K^+^ Currents
LTD: Long Term Depression
LTP: Long Term Potentiation
MAPK: Mitogen-activated Protein Kinase
mTOR: Mechanistic Target of Rapamycin
MWM: Morris Water Maze
NF-jB: Nuclear Factor-jB
NMDA: N-methyl-D-aspartate
NO: Nitric Oxide
NOX: NADPH Oxidases
Nrf-2: Nuclear Respiratory Factor 2
PME: Prenatal Melamine Exposure
PPF: Paired-pulse Facilitation
PSD95: Postsynaptic Density Protein 95
ROS: Reactive Oxygen Species
SC: Schaffer Collaterals
sEPSCs: Spontaneous Excitatory Postsynaptic Currents
SOD: Superoxide Dismutase
VGSCs: Voltage-gated Sodium Channels
